# Origins and consequences of hyperosmolar stress in retinal pigmented epithelial cells

**DOI:** 10.3389/fphys.2014.00199

**Published:** 2014-05-30

**Authors:** François Willermain, Sarah Libert, Elie Motulsky, Dany Salik, Laure Caspers, Jason Perret, Christine Delporte

**Affiliations:** ^1^Department of Ophthalmology, CHU Saint-Pierre and BrugmannBrussels, Belgium; ^2^I.R.I.B.H.M, Université Libre de BruxellesBrussels, Belgium; ^3^Laboratory of Pathophysiological and Nutritional Biochemistry, Department of Biochemistry, Université Libre de BruxellesBrussels, Belgium

**Keywords:** retinal pigmented epithelium, blood-retinal barrier, hyperosmolar stress, diabetes, age-related macular degeneration, uveitis, edema

## Abstract

The retinal pigmented epithelium (RPE) is composed of retinal pigmented epithelial cells joined by tight junctions and represents the outer blood-retinal barrier (BRB). The inner BRB is made of endothelial cells joined by tight junctions and glial extensions surrounding all the retinal blood vessels. One of the functions of the RPE is to maintain an osmotic transepithelial gradient created by ionic pumps and channels, avoiding paracellular flux. Under such physiological conditions, transcellular water movement follows the osmotic gradient and flows normally from the retina to the choroid through the RPE. Several diseases, such as diabetic retinopathy, are characterized by the BRB breakdown leading to leakage of solutes, proteins, and fluid from the retina and the choroid. The prevailing hypothesis explaining macular edema formation during diabetic retinopathy incriminates the inner BRB breakdown resulting in increased osmotic pressure leading in turn to massive water accumulation that can affect vision. Under these conditions, it has been hypothesized that RPE is likely to be exposed to hyperosmolar stress at its apical side. This review summarizes the origins and consequences of osmotic stress in the RPE. Ongoing and further research advances will clarify the mechanisms, at the molecular level, involved in the response of the RPE to osmotic stress and delineate potential novel therapeutic targets and tools.

## Introduction

**Figure 1:**
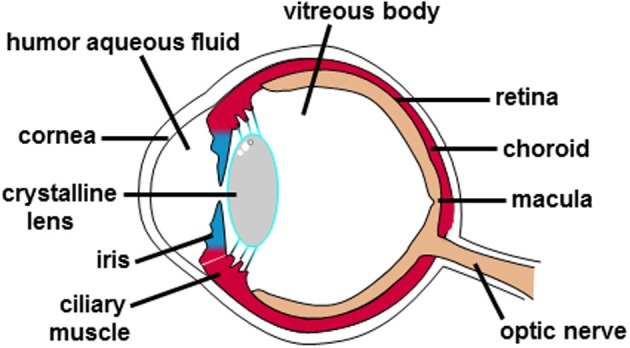
**Anatomical structure of the human eye**. The different anatomical entities of the human eye are indicated.

Upon stimulation by light, the eye transforms the light energy to ionic fluxes that are ultimately transmitted to the brain as action potentials. The anatomical structure of the eye is composed, from the front outer side to the back inner side, of several anatomical entities (Figure 1). Indeed, the cornea allows focusing light. The iris controls the amount of light reaching the back side of the eye. The crystalline lens (behind the pupil) further focuses light. The vitreous body maintains the shape of the posterior chamber of the eyeball and the retina in place. Finally, the retina converts light energy into chemical that are then transmitted as action potentials, via the optic nerve, to the optical cortex of the brain that in turn interprets and controls our sense of sight.

**Figure 2:**
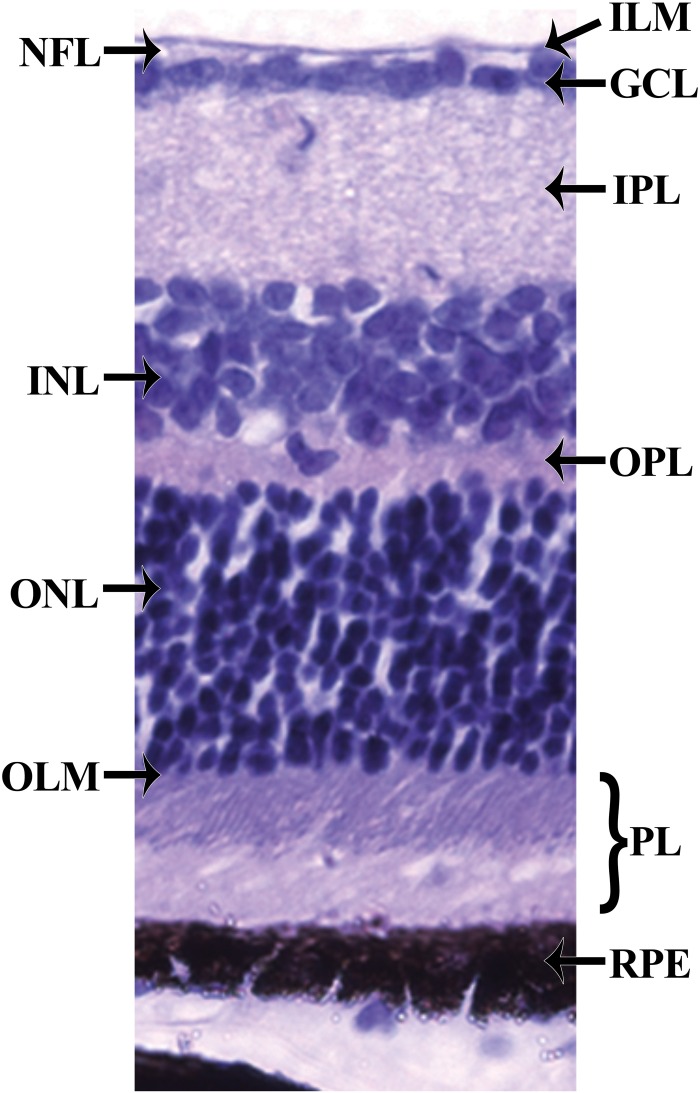
**Histology of the retina**. The retina can be divided into 10 layers including (1) the inner limiting membrane (ILM); (2) the nerve fiber layer (NFL); (3) the ganglion cell layer (GCL); (4) the inner plexiform layer (IPL); (5) the inner nuclear layer (INL); (6) the outer plexiform layer (OPL); (7) the outer nuclear layer (ONL); (8) the outer limiting membrane (OLM); (9) the photoreceptor layer (PL), and (10) the retinal pigmented epithelium (RPE) monolayer.

Histologically, the retina can be divided into 10 layers (Kolb et al., 1995) (Figure 2). From the inner to the outer part of the retina, these retinal layers are organized as follows. The first layer, the inner limiting membrane (ILM) is formed by the conical endfeet of the Müller cells and astrocytes. The second layer, the nerve fiber layer (NFL) consists of axons of ganglion cells, retinal vessels and glial cells. The third layer, the ganglion cell layer (GCL) predominantly contains the nucleus of ganglion cells, vessel cells, glial cells, and some displaced amacrine cells. The forth layer is the inner plexiform layer (IPL) where bipolar, amacrine, and ganglion cells interact. The fifth layer, the inner nuclear layer (INL) harbors the nuclei from bipolar, horizontal, amacrine, and Muller cells. The sixth layer is the outer plexiform layer (OPL) where photoreceptor cells connect with bipolar cells, and where horizontal cells interact closely with both photoreceptors and bipolar cells. The seventh layer, the outer nuclear layer (ONL) contains nuclei from photoreceptor cells. The eighth layer, the outer limiting membrane (OLM) is created by junctional complexes between adjacent Muller cells as well as between Muller and photoreceptor cells. The ninth layer, the photoreceptor layer (PL) contains tightly stacked cones and rods forming a pallisading layer of photoreceptors. The tenth layer results from tight junctional complexes between RPE cells forming a continuous RPE monolayer. Moreover, the RPE is separated from the choriocapillaris by the Bruch's membrane composed of 5 layers: the basement membrane of the choriocapillaries, an outer collagenous layer, a central elastic layer, an inner collagenous layer, and the basement membrane of the RPE. Basically, photons first pass through the neuroretina before activating the photoreceptor cells. A reduction in the circulating current triggers a change in the excitatory signaling to bipolar cells and subsequently ganglion cells. Bipolar and ganglion cell responses are modulated by horizontal and amacrine cells responses, respectively. Ganglion cell axons converge to form the optic nerve which carries the signal to the brain.

**Figure 3:**
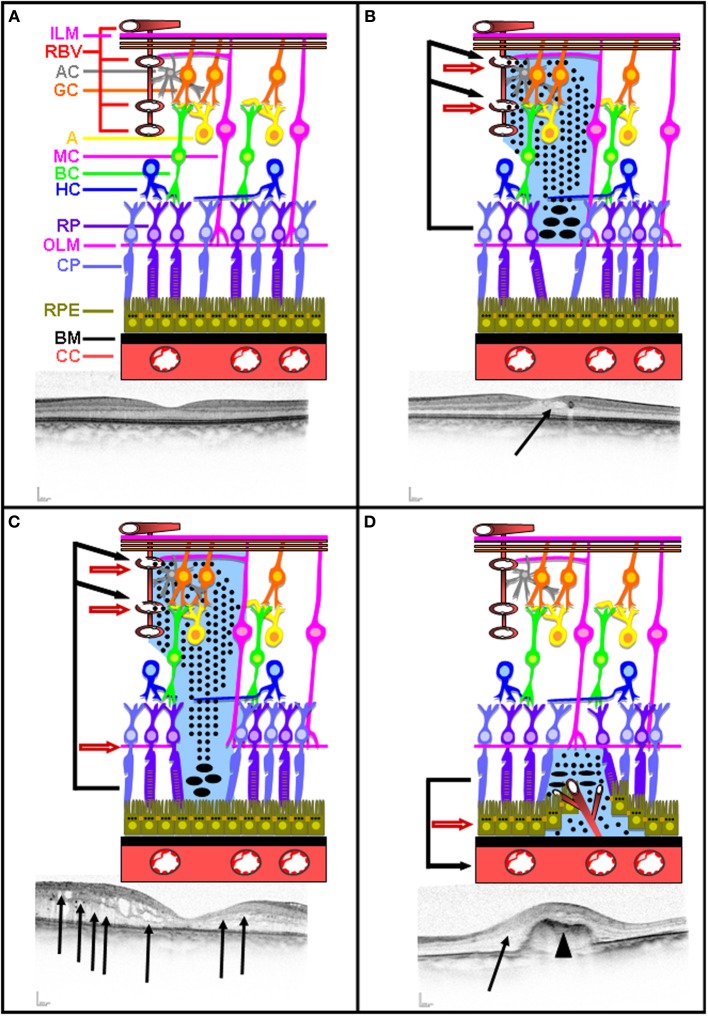
**Intact and disrupted BRB**. Schematic representation of intact inner and outer BRB representation illustrated with a corresponding OCT picture **(A)**. Schematic representation of inner BRB rupture without concomitant OLM rupture illustrated with a corresponding OCT picture **(B)**. Schematic representation of inner BRB rupture with concomitant OLM rupture illustrated with a corresponding OCT picture **(C)**. Schematic representation of outer BRB rupture following neovascularization occurring in AMD illustrated with a corresponding OCT picture **(D)**. Plain arrow indicates edema. Arrow head indicates neovascularization.

The retina is protected by a blood-retina barrier (BRB) composed of inner and outer components called inner and outer BRB (Figure 3A). The inner BRB is formed by tight junctions sealing the intercellular spaces between the non-fenestrated retinal endothelial cells covered by pericytes, astrocytes, and Müller cells end feet surrounding all retinal blood vessels. The outer BRB is composed of RPE cells joined by tight junctions. Maintenance of both inner and outer BRB relies on the integrity of tight junctions limiting thereby the paracellular flow. Tight junctions are formed by signaling, scaffolding and junctional adhesion proteins (such as claudin and occludin) (Hosoya and Tachikawa, 2012). Under physiological conditions, several membrane transporters ensures nutrient uptake across the BRB, and several ionic pumps and channels of the RPE cells create an osmotic transepithelial gradient. Under such physiological conditions, transcellular water movement follows the osmotic gradient and flows normally from the retina to the choroid through the aquaporin water channels in the RPE cell membranes.

Several ocular pathologies are characterized by a BRB breakdown. This leads to the leakage of solutes, proteins, and fluid from the retina. In addition to the well documented inner BRB breakdown resulting in protein leakage inside the retina, outer BRB breakdown resulting in protein leakage from the choroid into the retina has been more recently demonstrated (Xu et al., 2011). The concomitant increased osmotic pressure occurring in the retina leads to massive water accumulation and the development of macular edema that can alter sight. Under these conditions, it has been hypothesized that RPE is likely to be exposed to hyperosmolar stress at its apical side.

## Characteristics of the retinal pigmented epithelium

The retinal pigmented epithelium (RPE) is constituted of RPE cells assembled by tight junctions, forming a continuous epithelium monolayer. The RPE is characterized by a paracellular electrical resistance 10-fold higher than the transcellular resistance (Miller and Steinberg, 1977). This property defines RPE as the outer part of the BRB. The apical membrane of RPE exhibiting long microvilli faces the light-sensitive outer segments of the photoreceptors cells, while the basolateral membrane of the RPE faces the choriocapillaris (Bok, 1993; Marmorstein, 2001). Extracellular matrices, present on both sides of the RPE, allow interaction between the RPE and its adjacent tissues: the photoreceptors on the apical side, and the choriocapillaris on the basolateral side. The interphotoreceptor matrix (IPM), located at the apical side of the RPE, enables the exchange of signaling molecules, nutrients and metabolic end products between the RPE and the photoreceptors (Johnson and Hageman, 1991). Bruch's membrane, located at the basolateral side of the RPE, is a multilayered extracellular matrix structure allowing exchange of signaling molecules and nutrients between the RPE and the blood stream and between the RPE and the choroidal endothelial cells (Lerche, 1963).

## Functions of the retinal pigmented epithelium

The RPE fulfills multiple roles that are essential for visual function (Bok, 1993; Strauss, 2005); e.g.: (1) absorbing light energy which is focused onto the macula by the lens; (2) transporting nutrients such as glucose and vitamin A from the blood to the photoreceptors; (3) eliminating water (that accumulates in the subretinal space due to the metabolic activity of the photoreceptors) thanks to the presence of a transepithelial Cl^−^ transport from the subretinal space to the blood; (4) releasing K^+^ into the subretinal space to maintain constant excitability of the photoreceptors; (5) re-isomerizing all-trans retinal into 11-cis trans retinal; (6) phagocyting altered outer segments of photoreceptors; (7) secreting growth factors such as pigmented epithelium derived factor (PEDF) at the apical side and vascular endothelial growth factor (VEGF) at the basolateral side, and (8) maintaining the immune privilege of the eye due to its outer BRB function but also by interfering with signaling pathways coordinating the immune system.

## Involvement of the retinal pigmented epithelium in transcellular water movements

Multiple ion channels and transporters are involved in the functions of the RPE themselves, including in the regulation of their cell volume, and involvement in transcellular water secretion and absorption following light or dark exposure of the retina (Wimmers et al., 2007).

Upon light exposure of the photoreceptors, rhodopsin conformation changes into meta-rhodopsin II, leading to the activation of transducin, the subsequent activation of a phosphodiesterase hydrolyzing cGMP into 5'GMP, the inhibition of non-specific cation channels, and finally cell hyperpolarization.

Following photoreceptor hyperpolarization, cellular K^+^ efflux decreases and induces a reduction in subretinal K^+^ concentration (Strauss, 2005; Wimmers et al., 2007). This concomitantly inhibits the Na^+^-K^+^-2Cl^−^ co-transporter located at the apical side of the RPE and activates the inward rectifier K^+^ channels. K^+^ extrusion to the subretinal space via the inward rectifier K^+^ channels compensates for the light-induced decrease of subretinal K^+^ concentration. These events result in a decrease of the intracellular Cl^−^ and K^+^ concentrations, leading to cytosolic water extrusion and cell shrinkage. Consequently, intense metabolic activity of the photoreceptors produces a large accumulation of water in the subretinal space. This water is eliminated from the subretinal space into the choriocapillaris by the RPE and into the vitreous by Muller cells (absorption) in order to maintain the adherence of the RPE. Fluid absorption by the RPE involves distinct mechanisms occurring at the apical and basolateral membranes of the RPE (Wimmers et al., 2007). Interestingly, administration of loop diuretic, such as furosemide, to diabetic patients suffering from nephropathy and retinopathy has been shown to be beneficial for the treatment of DR as it induced a reduction in blood-retinal barrier leakage of fluorescein (Parving et al., 1989) and partial resolution of macular edema (Ciardella, 2014).

In the dark, the apical Na^+^/K^+^-ATPase generates a Na^+^ gradient from the extracellular to intracellular space. Besides, K^+^ recycling by photoreceptors leads to a higher subretinal K^+^ concentration than during light exposure of the retina, thereby stimulating the apical Na^+^-K^+^-2Cl^−^ co-transporter and concomitantly increasing K^+^ and Cl^−^ uptake. This increase in intracellular Cl^−^ provides the driving force for Cl^−^ extrusion at the basolateral membrane of the RPE through Ca^2+^-activated (Ca^2+^-dependent Cl^−^ channel and product of the vitelliform macular dystrophy 2 gene VMD2), volume-activated (volume regulated anion channels VRAC) and/or cAMP-activated (cystic fibrosis transmembrane regulator CFTR) ion channels (Wimmers et al., 2007).

In conclusion, the RPE fluid transport differs in the light- or dark-adapted eye, where the RPE cells are submitted to either cell shrinkage or swelling following water secretion or absorption, respectively. Detailed description and diagram summarizing the role of RPE in fluid transport can be found in previously published review papers (Strauss, 2005; Wimmers et al., 2007).

In epithelia, transcellular water transport flows through aquaporins, transmembrane proteins allowing the passage of water with respect to the osmotic gradient (Agre, 2004). So far, thirteen aquaporins (AQPs, named AQP0 to AQP12) have been identified. They have been classified, according to their permeability and structural characteristics, into classical aquaporins (AQP0, AQP1, AQP2, AQP4, AQP5, AQP6, AQP8; only permeable to water), aquaglyceroporins (AQP3, AQP7, AQP9, AQP10; permeable to water and small solute such as glycerol) and non-classical aquaporins (AQP11 and AQP12; whose permeability is still debated and display particular structural features) (Agre, 2004). AQP expression in RPE cells is variable among species and has been best documented in rat and human. Rat RPE cells have been shown to express AQP0 (Hollborn et al., 2011, 2012a), AQP1 (Hollborn et al., 2012a; Ortak et al., 2013; Köferl et al., 2014), AQP2 (Ortak et al., 2013), AQP3 (Hollborn et al., 2012a,b), AQP4 (Rehak et al., 2011; Ortak et al., 2013; Köferl et al., 2014), AQP5 (Hollborn et al., 2011, 2012a), AQP6 (Hollborn et al., 2012a; Ortak et al., 2013), AQP7 (Hollborn et al., 2012a), AQP8 (Hollborn et al., 2012a), AQP9 (Hollborn et al., 2011, 2012a; Ortak et al., 2013), AQP11 (Hollborn et al., 2011, 2012a). Human RPE cells have been shown to express several AQPs. The expression of AQP1 (Stamer et al., 2003) remains controversial (Hamann et al., 1998; Levin and Verkman, 2006) on human RPE cells. Human RPE cells have been shown to express AQP3 (Hollborn et al., 2012b) and AQP7 (Tran et al., 2013). Besides, AQP1, AQP3, AQP4, AQP5, AQP6, AQP7, AQP10, AQP11, and AQP12 have been shown to be expressed in hESC- and hiPSC-derived RPE cells (Juuti-Uusitalo et al., 2013). Therefore, AQPs are likely to be involved in RPE water secretion and absorption occurring following light and dark exposure of the retina, respectively.

## Origins of the hyperosmolar stress stimulating the retinal pigmented epithelium

Several ocular pathologies can result in the rupture of either the inner or the outer BRB, leading to the accumulation of water in the retina and the formation of edema. Depending on the origin, severity and duration of the edema in the macula, sight may be altered.

The major causes of inner BRB rupture are diabetic retinopathy (DR), retinal vein occlusion (RVO), and uveitis. DR represents the most frequent cause of blindness in adults in the USA (Congdon et al., 2003; Antonetti et al., 2012). In the USA, it is estimated that 86 and 40% of the patients suffering from type 1 or type 2 diabetes, respectively, develop DR. The physiopathology of DR is extremely complex. Indeed, chronic exposure to hyperglycemia and other causal risk factors initiate a cascade of biochemical and physiological modifications leading to microvascular damages and alteration of sight (Fong et al., 2004; Antonetti et al., 2012). The major factors involved in the pathogenesis of DR are sorbitol and advanced glycated-end products accumulation, oxidative stress, protein kinase C activation, inflammation, positive regulation of renin-angiotensin system and VEGF (Boscia, 2010). RVO, obstruction of the retinal venous system by thrombus formation, external compression, or alteration of the vein walls, is the second most frequent retinal vascular disease after DR affecting more than 16 millions people worldwide (Rogers et al., 2010). Several risks factors have been associated with RVO including hypertension, hyperlipidemia, diabetes, thrombophilia, hypercoagulation, systemic and inflammatory diseases, medications and ocular conditions (Jaulim et al., 2013). Infectious or non-infectious uveitis is an intraocular inflammation affecting principally the uvea, but also often the cornea, vitreous body, retina, and/or optical nerve (de Smet et al., 2011). The etiology, frequency, and characteristics of uveitis vary depending on the geographic localization (de Smet et al., 2011; Willermain et al., 2012). In a similar way, RD, RVO, and uveitis can cause the rupture of the inner BRB accompanied with or without the presence of concomitant rupture of the OLM, thereby causing neuroretinal edema, or neuroretinal and subretinal edema, respectively.

The prevailing hypothesis explaining macular edema formation in these diseases is: the rupture of the inner BRB without the presence of concomitant OLM rupture, damaged retinal vessels allow free protein diffusion deeper toward the outer retina (Figure 3B). Proteins accumulate at the nearby surface of the OLM, inducing protein precipitations thereby allowing additional continuous protein accumulation. This leads to an increase in osmotic pressure and a concomitant water accumulation creating an intra-retinal edema (Figure 3B).

Upon inner BRB rupture with the presence of concomitant OLM rupture, damaged retinal vessels allowfree protein diffusion toward the outer retina (Figure 3C). Proteins accumulate at the nearby surface of RPE, inducing protein precipitations and thereby allowing additional continuous protein accumulation and the persistence of the edema (Figure 3C).

Likewise, diabetes and ischemia also induce outer BRB breakdown as demonstrated by microscopic imaging assay using FITC-dextran in experimentally-induced diabetes and ischemia in rats (Xu et al., 2011).

Another major cause of outer BRB rupture is age-related macular degeneration (AMD). AMD represents a multifactorial disease affecting more than 50 millions people worldwide. Several risk factors have been associated with AMD including aging, genetic predisposition factors, smoking, obesity, and hypertension (Jager et al., 2008). AMD often leads to subretinal neovascularization, which induces the rupture of the RPE monolayer (Figure 3D). As a consequence of the outer BRB rupture, proteins originating from the choriocapillaris accumulate below the OLM and precipitate, thereby allowing additional continuous protein accumulation and the persistence of the edema. This leads to an increase in osmotic pressure and a concomitant water accumulation creating retinal edema (Figure 3D). In this particular case, RPE cells are also likely to be subjected to a hyperosmolar stress.

Altogether, these arguments strongly suggest that, at least for some forms of macular edema, RPE cells are indeed submitted to hyperosmotic stress.

## Consequences of hyperosmolar stress on the retinal pigmented epithelium

Cellular responses to hyperosmolar stress has extensively been studied in cells from the renal medulla, physiologically exposed to much higher concentrations of NaCl and urea than any other cell type of the human body, as well as in other cell types. However, the cellular responses of the RPE to hyperosmotic stress remain poorly understood as only very few studies have been performed so far.

Cell response to hyperosmolar stress, inducing cell shrinkage, results in regulatory volume increase (RVI) leading to cell volume recovery by swelling (Hoffmann et al., 2009). The main effectors involved in the early phases of RVI are the Na^+^-K^+^-2Cl^−^ cotransporters and Na^+^/H^+^ exchangers (Hoffmann et al., 2009). In addition, the hyperosmolar stress activates the transcription factor TonEBP/NFAT5 that transactivates several so called osmoprotective genes including taurine transporter, aldose reductase, betaine/GABA transporter, *myo*-inositol transporter and GPC (Burg et al., 2007). These genes are responsible for intracellular accumulation of organic osmolytes contributing to the late phases of RVI.

Interestingly, cellular responses to hyperosmolar stress is not restricted to osmoadaptation and include perturbing effects (such as cell cycle arrest, apoptosis, DNA damage, oxidative stress, mitochondrial depolarization, inhibition of transcription, and translation), cytoskeleton rearrangement, and modulation of stress proteins expression (such as Gadd45, p53, heat shock proteins, heme oxygenase 1) (Burg et al., 2007). The expression of heat shock proteins (HSPs), acting as molecular chaperones by preventing the accumulation of cytotoxic protein aggregates and assisting in correct folding of nascent and misfolded proteins, are increased in response to various stress factors, including hyperosmotic stress in the kidney (Burg et al., 2007) and other tissues (Brocker et al., 2012). Furthermore, TonEBP stimulates the transcription of HSP70 in response to hyperosmotic stress (Woo et al., 2002; Gogate et al., 2012). Increased HSPs levels have been detected in the retina of AMD and might be involved in this pathology (Kaarniranta et al., 2009). However, to our knowledge, increased HSPs levels have not been documented in RPE cells submitted to hyperosmolar stress. Induction of inflammatory cytokines is another important effect of hyperosmolarity that could be relevant in the context of macular edema (Neuhofer, 2010).

**Figure 4:**
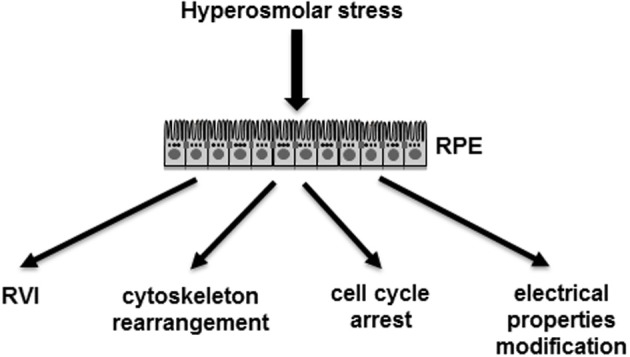
**Demonstrated effects of hyperosmolar stress on RPE cells**. RPE, retinal pigmented epithelium monolayer; RVI, regulated volume increase.

To date, the so far identified cellular responses of RPE cells to hyperosmolar stress remain quite limited (Figure 4). A first effect of hyperosmolar stress on RPE cells results in the accumulation of intracellular organic osmolytes. Indeed, in primary cultures of human RPE, hyperosmotic stress induces the expression and activity of aldose reductase (AR; EC 1.1.1.21), an enzyme catalyzing the transformation of glucose into sorbitol, and concomitantly increasing the intracellular sorbitol concentration serving as an osmolytes to protect cells from hyperosmotic-induced cell shrinkage (Lin et al., 1993; Sato et al., 1993; Henry et al., 2000).

Increased AR expression in response to hyperosmolar stress in RPE cells is likely to enhance DR, as AR is involved in adverse cellular effects during DR. Indeed, the potential beneficial effects of using specific AR inhibitors as potential treatment for DR are currently being investigated in a clinical trial (Obrosova and Kador, 2011). Besides, it has been shown very recently in retina from streptozotocin-induced diabetic mice that hyperglycemia induced TonEBP activation and that RNA silencing of TonEBP reduced AR expression and apoptotic cell death (Park et al., 2014). Therefore, it has been suggested that TonEBP reduction could represent an effective therapeutic strategy for the treatment of DR. Taurine, the most abundant retinal amino acid, which is essential for maintenance of retinal structure and function (Pasantes-Morales et al., 1972; Hayes et al., 1975), is also involved in the osmoregulation of the RPE cells. Indeed, increased taurine transporter activity (uptake) coupled with increased intracellular concentration of taurine occurred in primary culture of human RPE (Lin et al., 1993; Hillenkamp et al., 2004) and in a well-established human RPE cell line (ARPE-19) (El-Sherbeny et al., 2004) in response to hyperosmolar conditions. *Myo*-inositol represents another small organic solute involved in cell osmoregulation and its transport is ensured by a *myo*-inositol transporter (Burg et al., 2007). Both taurine and *myo*-inositol transporters activities are Na^+^ dependent (Reddy, 1973).

A second effect of hyperosmolar stress on RPE cells results in cytoskeleton rearrangement. Indeed, the expression of lysyl oxidase, an extracellular amine oxidase controlling the maturation of collagen and elastin, increased in response to hyperosmotic stress applied to the apical side of cultured rabbit RPE cells and accounted for collagen functionality (Omori et al., 2002).

A third effect of hyperosmolar stress, on the ARPE-19 human RPE-derived cell line, results in cell cycle arrest. Indeed, gene expression profiling in ARPE-19 cells submitted to hyperosmolar stress led to the identification of a subset of regulated genes involved in regulation of cellular proliferation, transcription from RNA polymerase II promoter and response to abiotic stimuli (Arsenijevic et al., 2013). Moreover, cell number, cell proliferation, and cell cycle phases were altered by hyperosmotic stress in ARPE-19 cells, while apoptosis and necrosis remained unaffected (Arsenijevic et al., 2013). In agreement with the decreased percentage of cells in G0/G1 and S phases and the increased percentage of cells in G2/M phase, hyperosmotic stress of ARPE-19 cells induced a decrease in cyclin B1 and D1 expression, and an activation of the p38-mitogen-activated protein kinase (Arsenijevic et al., 2013).

A fourth effect of hyperosmolar stress on RPE results in cell volume regulation. Hyperosmolar-induced shrinkage of human and frog RPE caused a RVI (Adorante and Miller, 1990; Civan et al., 1994). Frog RPE acted almost as ideal osmosensors as the steady-state changes in intracellular osmolarity closely reflected the retinal extracellular osmolarity changes (La Cour and Zeuthen, 1993). The hyperosmolar-induced upregulation of AQP3 (Hollborn et al., 2012a) and AQP5 (Hollborn et al., 2012b) and downregulation of AQP4 (Willermain et al., in press) in human RPE and ARPE-19 could contribute to transcellular water flow occurring during RVI. However, the roles of the multiple AQPs identified in RPE still remain to be addressed.

A fifth effect of hyperosmolar stress at the apical side of RPE results in the modification of its electrical properties. Transepithelial electrical resistance (TER) from the bovine RPE is slightly decreased by hyperosmolar stress when applied at the apical side of the epithelia (Orgül et al., 1993). In addition, hyperosmolar stress of the RPE induced an increase in the ocular standing potential (positive wave) (Yamada et al., 1987). Furthermore, the apical and basolateral membrane potentials of frog RPE were simultaneously hyperpolarized by hyperosmotic stress applied at either the apical or basolateral side of the epithelia (Mukoh et al., 1985). When hyperosmolar stress is applied at the apical side of the epithelia, the apical membrane potential is greater than the basolateral membrane potential, with an increased transepithelial potential (Mukoh et al., 1985). When hyperosmolar stress is applied at the basolateral side of the epithelia, it induces the hyperpolarization of the basolateral membrane potential, and simultaneously of the apical membrane potential to a lesser extent, thereby resulting in a reduction in transepithelial potential (Mukoh et al., 1985). In chick RPE, retinal hyperosmotic stress depolarized the basolateral membrane and increased the amplitude of the light-evoked c-wave of the electroretinogram, decreased basolateral membrane resistance and RPE membrane polarization during the c-wave (Shirao and Steinberg, 1987). While choroidal hyperosmotic stress led to the hyperpolarization of the basolateral membrane, a decrease in amplitude of the light-evoked c-wave, and an increase of the basolateral membrane resistance (Shirao and Steinberg, 1987).

Furthermore, epigenetic modifications, which can alter gene expression without permanent changes in DNA sequence, have recently emerged as playing roles in DR (Kowluru et al., 2013). DNA methylation, histone modification and miRNAs are considered to be the epigenetic modifications that can regulate gene expression (Kowluru et al., 2013). These epigenetic modifications, as well as oxidative stress and advanced glycation end products, have been shown to be responsible for long-term and persistent adverse effects of DR beyond the point when glycemic control has been achieved (Zhang et al., 2012). These persistent effects have been defined as “metabolic memory” (Zhang et al., 2012). It is tempting to speculate that, in addition to hyperglycemia, other stimuli including hyperosmolar stress are likely to induce metabolic memory in RPE cells. Further experiments will be required to test this new hypothesis.

## Conclusion

Many arguments strongly suggest that RPE cells are submitted to hyperosmolar stress during macular edema. Only few studies have thus far documented the cellular responses of the RPE cell monolayer to hyperosmolar stress. However, none have investigated the potential importance of hyperosmolar stress on RPE function closely involved in macular edema formation. Additional studies are therefore required to deepen our understanding of the molecular mechanisms involved in these cellular responses. More particularly, investigating the possible role of TonEBP/NFAT5 in the RPE cellular responses to hyperosmolar stress are warranted and are currently under investigation in our laboratory.

### Conflict of interest statement

The authors declare that the research was conducted in the absence of any commercial or financial relationships that could be construed as a potential conflict of interest.
